# Encouragement to submit manuscripts-suggestions from a Japanese Associate Editor of Acta Cirurgica Brasileira

**DOI:** 10.1590/s0102-865020200040000000

**Published:** 2020-06-02

**Affiliations:** I MD, PhD, Department of Organ Fabrication, Keio University School of Medicine, Tokyo, Japan.

**Keywords:** Medicine, Practice Guideline, Research, International Cooperation, Medical Journalism

## Abstract

Medicine can be broadly divided into two fields: clinical medicine that aims to save existing patients and basic medical research that aims to save the lives of future patients. In terms of disseminating basic medical research, medical journals play a vital role for physicians and scientists, as they enable them to share experiences. The author, who has been serving as an Associate Editor of the Brazilian journal Acta Cirurgica Brasileira over a long period of time, wishes to encourage physicians and researchers to submit their papers to medical journals. As we currently face the difficult battle against COVID-19 pandemic, physicians worldwide must team up and fight the virus for the safety of our future generations.

Currently, the COVID-19 pandemic has not only negatively affected the lives of infected patients, their families, and health care professionals but has also brought the world and its economy to a halt. Medical science can be broadly divided into practice that is oriented towards the treatment of current patients and research about future medical issues. In terms of the latter, international medical journals offer a means to share research findings.

I am Eiji Kobayashi, a Japanese surgeon and a researcher specializing in organ transplantation and regenerative medicine. I am also involved in the research and development of state-of-the-art medical devices. Ever since accepting an offer from Professor Edna Montero, I have been serving as an Associate Editor of the journal Acta Cirúrgica Brasileira for over 20 years, and to date, I am the only Japanese Associate Editor. Meanwhile, Acta Cirurgica Brasileira has grown to be an internationally recognized journal, and its impact factor has been gradually increasing, reaching almost 1.0 recently^1^.

A large number of scientists who are currently engaged in fighting the novel coronavirus may be finding their research activities significantly restricted. To protect ourselves from getting infected, we may need to publish our research findings by submitting our papers to journals while staying at home, and not while working at our affiliated institutions.

Brazil and Japan have a long history of interactions, such as cultural exchanges. In the context of surgeons, we must not forget Professor Masayuki Okumura who performed clinical small bowel transplantation at the University of Sao Paulo when organ transplantation therapy was in its early stages^2^. He was one of the global pioneers of clinical small bowel transplantation.

To many people, however, one of the most famous common aspects of our cultures may be soccer. In the old days, Kazuyoshi Miura, a soccer player who is still active in Japan, travelled alone to Brazil at the age of fifteen to become a professional soccer player there. Recently, a Japanese soccer player Keisuke Honda, who was born and raised in Japan, was reported to have joined a Brazilian club.

I have had opportunities to interact with Professor Edna Montero through the International Society for Experimental Microsurgery (ISEM) over the years. At the Conference of the Brazilian Society for the Development of Surgical Research (SOBRADPEC) held in Natal in 2005 (Fig. 1), I proposed a new concept of translational research that connected the basic research with the clinics^3^. This concept has become very popular in various surgical fields ever since^4^. The ISEM Executive Council Meeting 2010 in Sao Paulo that I attended as the president of the society created unforgettable memories (Fig. 2). In this congress, I showed a new trend of microsurgery^5^.

**Figure 1:**
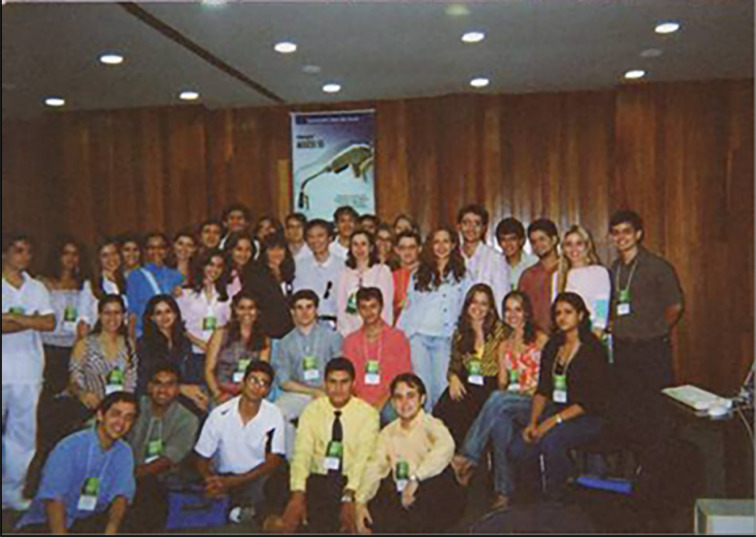
The Conference of the Brazilian Society for the Development of Surgical Research (SOBRADPEC) held in Natal in 2005 where I had opportunities to interact with young surgeons. The figure shows the young surgeons who attended the “Hands-on Seminar.”

**Figure 2:**
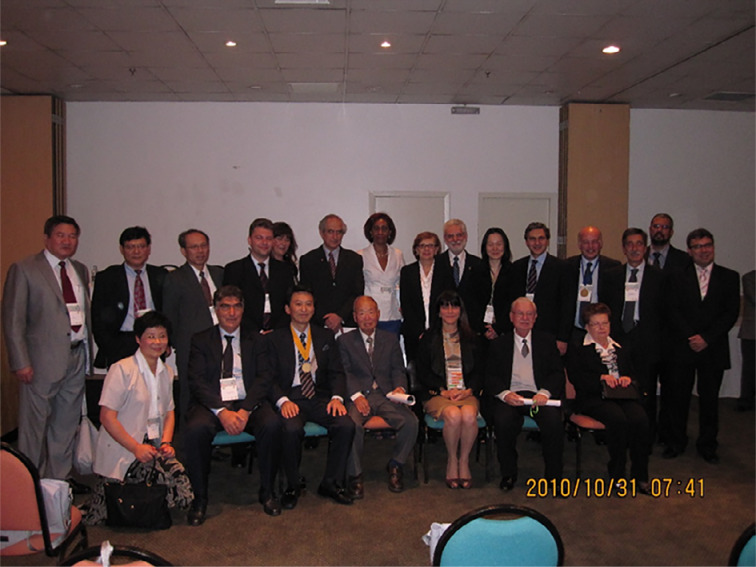
The Executive Council Meeting of ISEM 2010 in Sao Paulo. The late Professor Saul Goldenberg (front row, second from the right), the late Professor Sun Lee (front row, fourth from the right), Professor Edna Montero (front row, third from the right), Professor Murched O. Taha (front row, sixth from the right) and me (front row, fifth from the right).

To increase the journal's readership and to ensure its international success, I believe that we, the editors, are required to make every effort to review the submitted manuscripts carefully, to publish logically sound papers, and also to publish reviews that aim to describe the future of surgical medicine.

I sincerely hope that young surgeons of the next generation will make advances by submitting their manuscripts to our journal and become scientists who can think globally. Finally, I would like to suggest that we, the health care professionals, along with the patients affected by the severe COVID-19 and their family members, must team up and strive to fight against the virus.
